# A low coefficient of variation in hepatic triglyceride concentration in an inbred rat strain

**DOI:** 10.1186/s12944-020-01320-9

**Published:** 2020-06-16

**Authors:** Tomoko Shimoda, Shota Hori, Kenta Maegawa, Akari Takeuchi, Yeonmi Lee, Ga-Hyun Joe, Yasutake Tanaka, Hidehisa Shimizu, Satoshi Ishizuka

**Affiliations:** 1 Research Faculty of Agriculture, Hokkaido University, Kita-9, Nishi-9, Kita-ku, Sapporo, Hokkaido 060-8589 Japan; 2 Lee Gil Ya Cancer and Diabetes Institute, Gachon University, 155, Gaetbeol-ro, Yeonsu-gu, Incheon, 21999 Korea; 3 Research Faculty of Fisheries, Hokkaido University, 3-1-1, Minato-cho, Hakodate, Hokkaido 041-8611 Japan; 4 Faculty of Agriculture, Graduate School, Kyushu University, 744, Motooka, Nishi-ku, Fukuoka, Fukuoka 819-0395 Japan; 5 Institute of Agricultural and Life Science, Academic Assembly, Shimane University, 1060, Nishikawatsu-cho, Matsue, Shimane 690-8504 Japan

**Keywords:** Coefficient of variation, Cholesterol, Inbred, Liver, Triglycerides

## Abstract

**Background:**

Inbred strains are characterized by less genetic variation, which suggests usefulness of inbred strains for evaluations of various parameters. In this study, experimental reproducibility in several parameters was compared between an outbred Wistar rat and Wistar King A Hokkaido (WKAH/HkmSlc) rat, the inbred strain that is originated from Wistar rats.

**Methods:**

Difference of variations was investigated in parameters of body compositions and liver functions such as body weight, liver weight, liver triglycerides (TG), liver cholesterol and plasma alanine aminotransferase activity (ALT) between WKAH rats and outbred Wistar rats by using the coefficient of variation (CV).

**Results:**

There was no difference in the CVs of body weight and relative liver weight between WKAH and Wistar rats. The CVs of body weight and relative liver weight were below 10% in both WKAH and Wistar rats. The CVs of TG, cholesterol, and ALT in Wistar rats were between 30 and 40%, whereas those in WKAH rats were between 10 and 25%. A low CV level of TG was observed in WKAH rats compared to that in Wistar rats regardless of the duration of the experimental period in those rat strains.

**Conclusion:**

The low CV values in metabolic parameters involved in liver functions in the inbred rats suggested an advantage of using inbred rather than outbred rats for the evaluation of liver lipid metabolism.

## Background

In vivo experiments, particularly those using rodents, contribute greatly to understand the pathophysiology of humans. Inbred strains and outbred stocks are the two major classes of laboratory rodents [1]. Inbred strains are generally characterized by genetic uniformity, which results in less phenotypic variation in response to toxic or pharmacologic stimuli compared with outbred stocks [2]. In contrast, outbred stocks are characterized by being genetically variable. This suggests that the use of inbred strains improves experimental reproducibility with fewer animals. Outbred populations differ from randomly bred ones in that they are systematically bred to maintain the maximal genetic heterogeneity [3].

Recently, utilization of inbred strains in research has been proposed based on genetic stability [4], but difference of variation in metabolic parameters was still unclear among inbred and outbred animals. The use of outbred animals is prevailing in many fields of in vivo research. Considering the advantages of using inbred rodents, it is necessary to investigate variation in the parameters of interest between inbred and outbred rodents [5]. In this study, the coefficient of variation (CV) in several sets of experiments was compared in body weight, relative liver weight, and metabolic parameters between outbred Wistar rats and Wistar King A Hokkaido (WKAH) rats, an inbred strain of Wistar rats.

## Methods

### Animals

Three-week-old Wistar male rats (Slc:Wistar, Japan SLC, Inc., Shizuoka, Japan) and three-week-old Wistar King A Hokkaido male rats (WKAH/HkmSlc, Japan SLC) (NBRP Rat No: 0154) were used as an outbred stock and an inbred strain, respectively. They were housed in an air-conditioned room at 22 ± 2 °C with 55 ± 5% humidity, and a light period from 8:00 to 20:00. The rats were housed individually in wire-bottomed cages and allowed ad libitum access to food and water. They were acclimated to a diet based on the American Institute of Nutrition (AIN)-93G formulation [6]. At the end of the experimental period, the rats were anesthetized with sodium pentobarbital (50 mg/kg) and exsanguinated by blood withdrawal. The plasma and liver were dissected and stored at − 80 °C until analysis.

### Biochemical parameters

Alanine aminotransferase (ALT) in the plasma was measured using a transaminase CII-test Wako kit (Wako Pure Chemical Corporation, Osaka, Japan). Liver lipids were extracted using chloroform and methanol (2:1, v/v). The extracts were evaporated and dissolved in isopropanol and were used to measure triglyceride (TG) and cholesterol in the liver by using the Triglyceride E-test Wako and Cholesterol E-test Wako (Wako), respectively.

### Calculation of CVs

CV is the proportion of the standard deviation in the mean value. Several sets of experimental data were used in rats fed with the AIN-93G-based diet. In study I, Wistar rats were maintained for 6 weeks (6 rats per group and 4 groups). In study II, WKAH rats were maintained for 2 weeks (6 rats per group and 4 groups). In study III, WKAH rats were maintained for 13 weeks (10–12 rats per group and 4 groups). CVs were calculated from the values such as body weight (g), relative liver weight (g/kg body weight), liver TG (mg/g liver), liver cholesterol (mg/g liver) and plasma ALT (IU/L) in every group as mentioned above, which resulted in the number of CVs in each study was four (group) in each parameter (*n* = 4). Those calculated CVs were used for the statistical analysis as follows.

### Statistical analysis

The Tukey-Kramer test was used to compare CVs in each parameter among studies I, II, and III. Pearson correlation analysis were performed between CV of liver TG concentration and of liver cholesterol concentration and between CV of body weight and of relative liver weight. JMP software (ver. 14.0; SAS Institute, Caty, NC, USA) was used for the Tukey-Kramer test and Pearson correlation analysis. A probability of less than 0.05 was considered as significant. To determine an adequate sample size to identify significant differences in hepatic iron concentration, a power analysis was performed using G*Power (version 3.1.9.4) [7] based on the experimental design. Based on an α probability of 0.05 and a power of 0.80, the effect size was estimated using the results from preliminary studies (unpublished results). The required sample (number of group) size was from three to nine per study.

## Results

**Fig. 1:**
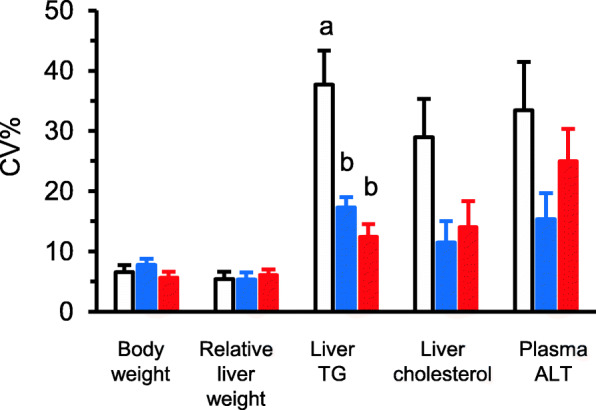
Comparison of CVs of several parameters in outbred Wistar rats (study I) and inbred WKAH rats (study II for 2 weeks and study III for 13 weeks). Bars represent CVs in outbred Wistar (open bar) for 6 weeks and inbred WKAH (blue bars for 2 weeks; study 2 and red bars for 13 weeks). Values not sharing the same letter are significantly different (Tukery-Kramer test, *P* < 0.05, *n* = 4)

The CVs of body weight and relative liver weight were around 5 to 10%, whereas those of biochemical parameters were from 10 to 40% (Fig. 1). There was no significant difference in the CVs of body weight, liver weight, cholesterol, and ALT among the studies. The CV in TG was significantly lower in the study II and III (WKAH for 2 weeks and 13 weeks) than in the study I (Wistar rats for 6 weeks) (Fig. 1). In other biochemical parameters such as cholesterol and ALT, the CVs in the Wistar rats tended to be a higher than those of WKAH rats regardless of the duration of the experimental period. These results showed that the CVs of body and relative liver weight were below 10%, regardless of the genetic background of the rats.

**Fig. 2:**
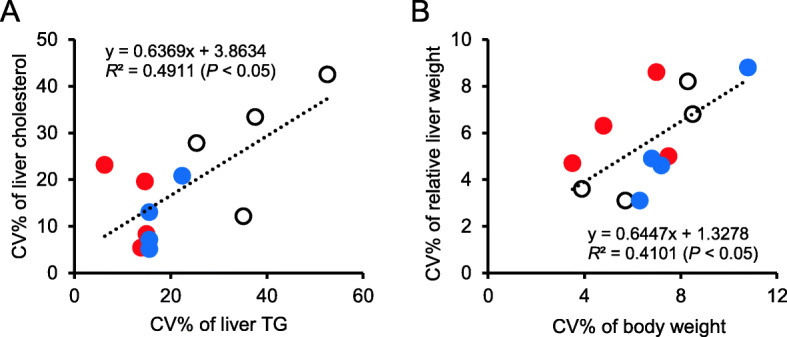
Pearson correlation between the CVs of parameters determined. **a** CVs of liver TG and liver cholesterol in outbred Wistar rats (study I) and inbred WKAH rats (study II for 2 weeks and study III for 13 weeks). **b** CV of body weight and CV of relative liver weight in outbred Wistar rats (study I) and inbred WKAH rats (study II for 2 weeks and study III for 13 weeks). Each spot shows the individual CVs in each group of Wistar rats (open circle, 6 weeks) or WKAH rats (blue circle, 2 weeks; red circle, 13 weeks)

The CV of liver TG correlated with that of liver cholesterol (*R*^2^ = 0.49, *P* < 0.05) (Fig. 2a). There was a positive correlation (*R*^2^ = 0.41, *P* < 0.05) between the CVs of body weight and relative liver weight (Fig. 2b). In WKAH rats, the CV plots of TG and cholesterol were accumulated at relatively low levels (Fig. 2a), whereas those of Wistar rats were scattered at higher levels. Such a trend was not observed in the CVs of body weight and relative liver weight (Fig. 2b).

## Discussion

The CV is frequently used to qualify the accuracy of measurement systems [8]. Since the CV is an indicator of variation, it can evaluate deviation in any aspect of biological parameters. In this study, alteration of CVs was assessed in the weight and metabolism that are frequently used in experimental nutrition research among WKAH (2 weeks and 13 weeks) and Wistar rats. Basically, the CVs of weight (body weight and relative liver weight) were relatively small (below 10%) in all the rats, whereas those of metabolic parameters such as TG, cholesterol, and ALT were above 10%. Notably, the lower value in the CV of liver TG in the inbred than in outbred rats suggests that the stability of the genetic background in inbred rats avoided the fluctuation in the parameters.

In literature, the CVs of body weight of various inbred mouse strains range from 4.2 to 10.1% [9]. Similarly, the CV of body weight in the present study falls within the same range [9] regardless of the genetic background. In the case of metabolic parameters, much higher CVs were reported. For example, CVs in plasma amino acid concentrations were from 3 to 28% and the majority was around 15% in clinical samples [10]. The CV of ^18^fluoro-2-deoxy-D-glucose incorporation in human pulmonary cancer cells is around 31.9% [11]. In the case of plasma glucose, the CV ranges from 34 to 51% in overnight-fasted monkeys [12]. Additionally, a high CV value in γ-aminobutyric acid concentration makes it difficult to detect the difference between control and treatment groups in the metabolomic analysis of ^1^H-NMR [13], indicating the advantage of a lower CV for detecting differences in metabolite concentrations among treatments.

The prevention of obesity and metabolic disorders is necessary to reduce the risk of noncommunicable diseases [14]. Studies in experimental animals have provided valuable information about biological processes in the development of metabolic diseases, such as obesity, type 2 diabetes, and non-alcoholic fatty liver diseases [15, 16], and enabled the establishment of various metabolic markers for the prevention and diagnosis of these disorders. The present study suggests the importance of using inbred animals with lower CV values of metabolites for accurate evaluation of metabolic alterations.

In outbred stocks, it is sometimes difficult to detect a significant difference in biochemical parameters among treatments in toxicology [1]. In contrast, such differences can be detected easily in inbred strains [2] due to stability of genetic background [3]. The CVs in all the parameters in inbred strains are not necessarily less than those in outbred stocks. The present study also showed that the CVs of weight parameters were almost comparable between WKAH and Wistar rats. In nutritional science, rats are preferentially used because their body size enables extraction of sufficient quantities of organs, biological fluids such as blood or intestinal contents, etc. Further, a PubMed search revealed that 80% of inbred strains used are mice, while 10% are outbred rats [5]. This observation suggests that researchers do not recognize the advantage of using inbred strains to detect a difference in metabolic parameters among treatments, especially in rat studies.

To obtain a significant difference between the values in biological parameters, CVs of about 7% in each value are generally required [17]. Since the CVs of body weight and relative liver weight fell in this range (Fig. 1), a change in these body composition parameters can be easily detected even in outbred stocks. In other words, detection of significant difference in metabolic parameters such as TG, cholesterol, and ALT, would be difficult, especially in outbred stocks.

This study suggests an advantage of inbred rats to detect differences especially in several metabolic parameters in rats, which contributes to reduction in number of animals in an experiment. It should be noted that an appropriate sample size depends on the parameters of interest.

## Conclusion

The present study demonstrated that the CV of liver TG in inbred WKAH rats was significantly less than that in outbred Wistar rats. The results suggest that the use of inbred rats can help in the accurate evaluation of metabolic parameters in an animal experiment. To find relevant parameters in animal studies is expected to contribute to elucidation of significant connection between parameters in a clinical study although there might be much broader deviations of metabolic parameters in clinical studies.

## Data Availability

The datasets analyzed during the current study are included in the manuscript.

## Abbreviations

ALT: alanine aminotransferase
CV: coefficient of variation
TG: triglyceride
